# Improvement in the Sequential Extraction of Phycobiliproteins from *Arthrospira platensis* Using Green Technologies

**DOI:** 10.3390/life12111896

**Published:** 2022-11-15

**Authors:** Wanida Pan-utai, Siriluck Iamtham, Sumitra Boonbumrung, Juta Mookdasanit

**Affiliations:** 1 Department of Applied Microbiology, Institute of Food Research and Product Development, Kasetsart University, Chatuchak, Bangkok 10900, Thailand; 2 Department of Science, Faculty of Liberal Arts and Science, Kasetsart University, Kamphaeng Saen, Nakhon Pathom 73140, Thailand; 3 Center for Agricultural Biotechnology, Kasetsart University, Kamphaeng Saen Campus, Nakhon Pathom 73140, Thailand; 4 Center of Excellence on Agricultural Biotechnology—(AG-BIO/PERDO-CHE), Bangkok 10900, Thailand; 5 Department of Food Chemistry and Physics, Institute of Food Research and Product Development, Kasetsart University, Chatuchak, Bangkok 10900, Thailand; 6 Department of Fishery Products, Faculty of Fisheries, Kasetsart University, Chatuchak, Bangkok 10900, Thailand

**Keywords:** *Spirulina*, *Arthrospira*, phycobiliproteins, pigments, supercritical fluid extraction, green technology

## Abstract

*Arthrospira platensis* (commercially known as *Spirulina*) is an excellent source of phycobiliproteins, especially C-phycocyanin. Phycobiliproteins are significant bioactive compounds with useful biological applications. The extraction process plays a significant role in downstream microalga production and utilisation. The important pigments found in *A. platensis* include chlorophyll and carotenoids as nonpolar pigments and phycobiliproteins as polar pigments. Supercritical fluid extraction (SFE) as a green extraction technology for the high-value metabolites of microalgae has potential for trends in food and human health. The nonpolar bioactive compounds, chlorophyll and carotenoids of *A. platensis,* were primarily separated using supercritical carbon dioxide (SC-CO_2_) solvent-free fluid extraction pressure; the temperature and ethanol as cosolvent conditions were compared. The residue from the *A. platensis* cells was subjected to phycobiliprotein extraction. The phosphate and water extraction of *A. platensis* SFE residue were compared to evaluate phycobiliprotein extraction. The SFE results exhibited higher pressure (350 bar) and temperature extraction (50 °C) with ethanol-free extraction and increased nonpolar pigment. Phycobiliprotein yield was obtained from *A. platensis* SFE residue by ethanol-free buffer extraction as a suitable process with antioxidant properties. The C-phycocyanin was isolated and enhanced to 0.7 purity as food grade. This developed method can be used as a guideline and applied as a sustainable process for important pigment extraction from *Arthrospira* microalgae.

## 1. Introduction

*Arthrospira platensis* (commercially known as *Spirulina*) is a filamentous cyanobacterium and blue-green microalga commonly supplemented in functional foods [1,2], nutraceuticals [3,4] and animal feed [5] and is also used for biofuel production [6]. *A. platensis* can utilise carbon dioxide as a nutrient source for biomass production [7]. *Arthrospira* biomass is a rich source of both macro and micronutrients and is used as a food and dietary supplement due to its therapeutic properties such as antioxidant and anti-inflammatory activities [1,8,9]. *Arthrospira* is also a good source of natural proteins, carbohydrates, lipids, vitamins, enzymes and pigments including chlorophyll, carotenoids and phycocyanin [10,11]. *Arthrospira* can be cultivated in open systems on a large scale for high biomass production [12], making it a significant and interesting natural source for valuable biosubstances and functional ingredients. The WHO has designated *Arthrospira* as a superfood due to its potentially rich source of chemicals with biological activities that can also be employed as functional components. Recently, increased knowledge of the properties of the health-boosting nutrients and nutraceuticals has improved lifestyles. As a result, *Arthrospira* microalgae are now attracting increased interest in the nutritional and food science fields [13].

*Arthrospira* is also a valuable source of natural pigments including chlorophyll, carotenoids and phycobiliprotein, especially C-phycocyanin (C-PC) [11,14]. The expanded use of *Arthrospira* has coincided with increased consumer knowledge of the value of natural colourants and their advantages in terms of nutrition, pharmacology and health. As a result, more natural colours are being used, with spirulina serving as a popular source of these pigments, particularly in the food and cosmetic sectors [15]. Chlorophyll is one of the main photosynthetic pigments in natural systems with a role in the photosynthetic process of absorbing light, transferring energy and transporting electrons. Chlorophyll is also utilised in several industries for its photophysical and photochemical capabilities, such as food colouring and optically active centres for luminescent solar concentrators [16,17]. Microalgae are the main source of valuable compounds obtained through photosynthesis [18]. Carotenoids are insoluble in water and found in *Arthrospira* [19]. The bioavailability of carotenoids from *Arthrospira* has promising potential as a source of provitamin A which has high antioxidant activity and ameliorates cardiovascular disorders, cancer and anti-aging activity [20]. Natural carotenoids are used in industrial applications as food colourants, feed additives, cosmetics and pharmaceuticals [21].

Phycobiliproteins (PBPs) are the light-harvesting pigment proteins of phycobilisomes (antenna complexes), which act as photosynthetic accessory pigments in cyanobacteria [22]. PBPs can be divided into three groups on the basis of their absorption characteristics as C-phycocyanin (C-PC, blue pigment), allophycocyanin (APC, light-blue pigment) and phycoerythrin (PE, red pigment) [23,24]. Phycobiliproteins are used for various diagnostic scientific research and therapeutic purposes [25] and mainly consist of C-phycocyanin (C-PC) which is a well-known pigment with antioxidant, anti-inflammatory and anticarcinogenic activities [26]. Phycocyanin serves as the main photosynthetic pigment autotroph of *Arthrospira* [27]. Phycobiliproteins can be used as safe alternatives to synthetic colours, which are frequently poisonous or otherwise dangerous in food, cosmetics and pharmaceutical products [28]. Phycobiliproteins can be extracted using physical and chemically assisted methods; however, various aspects such as organism composition, stability and cell-wall resistance affect the choice of approach [29]. The biosynthetic recovery output of PBPs from biomass should also be considered and various levels of purity are necessary depending on how the PBPs will be used [30]. The primary phycobiliprotein in most blue-green algae is C-phycocyanin (C-PC) [31]. Phycobiliproteins have a market value of USD 5000–33,000 g^−1^ depending on quality as a natural pigment in the food, cosmetic, medical and biotechnology sectors [32]. The purity grade of phycobiliprotein has a significant impact on its commercial market value [33], with an extract purity of phycobiliprotein at 0.56–0.70 considered as food grade [34], 1.5 as cosmetic grade, 3.9 as reactive grade and greater than 4.0 as analytical grade [35]. Currently, several technologies exist for the commercial extraction of pigments from algae. The colours released from the ruptured cell wall of microalgae can be extracted using organic solvent extraction [36], pressurised solvent extraction [37], ionic liquid extraction, high-pressure homogenisation, ultrasonication [38] and supercritical carbon dioxide fluid extraction [39]. *A. platensis* is a photosynthetic multicellular blue-green microalga that is cultivated on a large scale for the commercial processing of biomass and bio-products [40]. Microalgae are becoming increasingly important, particularly for their composition because they contain high-value substances such as carotenoids, chlorophylls and phycobiliproteins [41]. Therefore, efficient methods for obtaining pigments and bio-products from microalgae are needed. These bioactive chemicals can be successfully extracted using SC-CO_2_ extraction as an environmentally friendly method [42].

Supercritical carbon dioxide (SC-CO_2_) fluid extraction is a green technology aimed at replacing organic solvent extraction [43]. The supercritical fluid extraction (SFE) approach is used by a variety of sectors to extract valuable bioactive chemicals [44]. The SFE technique has been widely employed as a separation technology in the food processing and pharmaceutical sectors for efficient and selective component extraction. SC-CO_2_ fluid extraction has evolved as a more environmentally friendly method than traditional petroleum-based solvent extraction procedures. SC-CO_2_ is a solvent that is widely used in SFE [45]. The special properties of SC-CO_2_ make it appealing for isolating essential oils, neutral lipids, flavours, perfumes, antioxidants and pigments from both terrestrial and non-terrestrial biomasses [46]. Most nonpolar solutes can be separated by SC-CO_2_ since it is lipophilic. In comparison to solvent extraction, CO_2_ separation is straightforward and leaves no residues in the extract [47]. Utilisation of the SC-CO_2_ approach focuses on the extraction of hydrophobic antioxidant chemicals [48]. A small amount of cosolvents can induce microalgal cells to expand, allowing fast mass transfer of analytes from the matrix [49]. The SC-CO_2_ extraction technique uses pressures and temperatures greater than the CO_2_ critical point [50]. Several studies have employed the SC-CO_2_ extraction process to purify active components (carotenoids and linolenic acid), oil and caffeic acid from microalgae [51,52,53]. SFE is a novel environmentally friendly technical method employed in the food and pharmaceutical industries to avoid the harmful organic solvents that use CO_2_ above its critical temperature and pressure points [54]. The addition of cosolvents induces the expansion of microalgal cells, allowing rapid mass transfer of analytes from the matrix. Carbon dioxide is the most commonly used SFE solvent in the pharmaceutical sector and is classed as safe by the USFDA [55]. The methods and processes of bioactive substance extraction have been improved to maximise the utilisation of biosubstances from microalgae. Nowadays, SFE is a popular green technology to extract nonpolar pigments from microalgae.

*Arthrospira platensis* is one of the main sources of natural commercial phycobiliproteins, especially C-phycocyanin. Therefore, the objectives of this study were to evaluate the efficiency of (i) extraction processes for nonpolar pigments: chlorophyll and carotenoids, using SFE as a green technology, before water-soluble phycobiliprotein extraction from *Arthrospira* biomass and (ii) evaluation of the improvement in the sequential phycobiliprotein extraction of cell biomass residues after SFE by comparing phosphate and water extraction with ultrasound-assisted extraction.

## 2. Materials and Methods

### 2.1. Microalgal Production

*Arthrospira platensis* IFRPD 1182 microalgae were prepared by the Institute of Food Research and Product Development, Kasetsart University, Thailand. The starter culture was maintained and prepared in Zarrouk medium [56] composed of (per litre) 16.8 g NaHCO_3_, 2.5 g NaNO3, 0.5 g K_2_HPO_4_, 1.0 g K_2_SO_4_, 1.0 g NaCl, 0.2 g Mg_2_SO_4_·7H_2_O 0.04 g CaCl_2_·7H_2_O, 0.01 g FeSO_4_·7H_2_O and 0.08 g EDTA. One millilitre each of vitamin A_5_ and B_5_ micronutrients was added into the medium. The micronutrient solution of A_5_ was composed of (per litre) 2.86 g H_3_BO_3_, 1.81 g MnCl_2_·4H_2_O, 0.22 g ZnSO_4_·7H_2_O, 0.08 g CuSO_4_·5H_2_O and 0.01 g MoO_3_. The B_5_ micronutrient solution was composed of (per litre) 22.9 mg NH_4_VO_3_, 96.0 mg K_3_Cr_2_(SO_4_)_4_·24H_2_O, 47.8 mg NiSO_4_·7H_2_O, 17.9 mg Na_2_WO_3_, 44.0 mg Co(NO_3_)_2_·6H_2_O and 40 mg Ti_2_(SO_4_)_3_. *A. platensis* was cultured and incubated in chamber equipment with temperature controlled at 30 °C [57]. Light intensity was controlled at a photon flux density of 162 µmol·m^−2^·s^−1^ using fluorescent 18 W daylight lamps with a 16 h/8 h light/dark cycle. Air mixed with 2% (*v*/*v*) CO_2_ at 0.67 vvm was added via continuous bubbling through a PTFE membrane filter. The *A. platensis* starter was grown for 7–14 days until reaching the log phase and then used at 10% (*v*/*v*) for biomass production. *A. platensis* was produced in Zarrouk medium in 200 L working volume in 500 L raceway ponds with a paddle wheel operated at 15 rpm. Average light photon flux density was 471 µmol·m^−2^·s^−1^ during open pond production with batch cultivation. The biomass of *A. platensis* was grown to the exponential phase for 15–20 days, with biomass concentration reaching 1 g·L^−1^. Cells were harvested using a 60 µm nylon membrane filter and washed with clean tap water until no residue remained in the culture medium. Then, the harvested cells were oven-dried at 65 °C for 4–6 h in a hot air oven. The oven-dried biomass of *A. platensis* was milled to 0.5 mm sample size using a mill grinder (ZM-1, Retsch, Haan, Germany) for use in the experiments.

### 2.2. Chlorophyll and Carotenoid Pigments Using Supercritical Fluid Extraction

**Figure 1:**
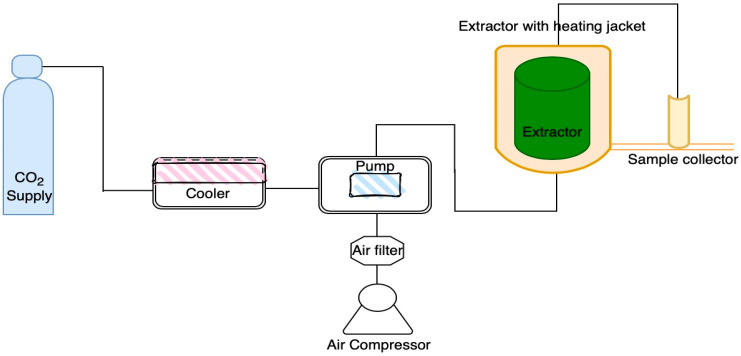
Overview of the SC-CO_2_ system.

Oven-dried *A. platensis* microalgal biomass was investigated for pigments extracted using SFE as a green technology. The chlorophyll and carotenoid pigments (nonpolar bioactive compounds) in *A. platensis* were extracted using an SC-CO_2_ pilot unit with a helix SFE System (Applied Separations Inc., Allentown, PA, USA). An overview of the SC-CO_2_ system is shown in Figure 1. The system included a solvent and carbon dioxide pump, a back-pressure regulator (BPR), a 1 L extractor vessel enclosed in a heating jacket, a pressure transmitter and a sample collector. The 5 g dry weight of *A. platensis* oven-dried biomass was added to a high-pressure stainless-steel extractor vessel. The experiments were performed with and without ethanol at 10% (*w*/*w*) of samples as the cosolvent. Polypropylene wool was used to mediate the inlet and outlet of the vessel. Static extraction was performed for 60 min, followed by dynamic extraction for 240 min, under various conditions of pressure at 250 and 350 bar with temperatures of 40 and 50 °C. Two main extraction experiments were compared: with and without ethanol as a cosolvent during SFE extraction (Table 1). Optimal conditions were cited from previous studies [58,59]. The flow rate was controlled at 3 litres per minute (LPM). The pigments were extracted and collected into the sample collector. All experiments were performed in duplicate. The extracted samples were kept in the dark at −20 °C for analysis. The residues of *A. platensis* biomass after nonpolar pigment extraction in the extractor were collected for sequential phycobiliprotein extraction.

### 2.3. Sequential Phycobiliprotein Extraction

*A. platensis* biomass residues after each SFE experiment (in extractor) were sequentially extracted for phycobiliproteins (PBPs), whereas oven-dried *A. platensis* biomass without SFE was used as the control. PBP extraction was performed using a biomass concentration of 0.02 g·mL^−1^ in 0.01 M phosphate extraction (0.01 M PB, pH 7.0) and water extraction (distilled water). The experiments were performed using ultrasonic-assisted extraction at frequency 35 kHz and power 320 W (DT 100H, Bandelin, Germany), for 30 min [60]. Temperature was maintained at around 25 °C by ice addition in an ultrasonic bath. The samples were incubated in the dark at 25 °C for 24 h and crude phycobiliproteins were collected from the mixtures by centrifugation at 3461× *g* for 10 min (EBA 200, Hettich, Tuttlingen, Germany). All experiments were performed in triplicate.

### 2.4. C-Phycocyanin Isolation

Crude phycobiliproteins were purified and concentrated using ultrafiltration with molecular weight cut-off (MWCO) of 100 kDa (Amicon Ultra-15 Centrifugal Filter Unit, Millipore, Merck, Darmstadt, Germany). The C-phycocyanin isolate was collected after centrifuging at 5000× *g* for 10 min at 20 °C (Model 6000, Kubota, Tokyo, Japan) for further analysis. All experiments were performed in triplicate.

### 2.5. Carotenoid and Chlorophyll Determination

Optical densities of the SC-CO_2_ fluid extracted samples were measured at 470, 645 and 662 nm using a UV–Vis spectrophotometer (SP-8001, UV–Vis Spectrophotometer, Metertech, Taiwan), with 100 (% *v*/*v*) acetone set as the blank. Total carotenoid and chlorophyll concentrations were calculated using the following equations [61]:

(1) $$\mathrm{Chlorophyll}\;a\;\left(\mathrm{mg}\cdot {\mathrm{mL}}^{-1}\right)=11.75{\mathrm{OD}}_{662}-2.350{\mathrm{OD}}_{645}.$$

(2) $$\mathrm{Chlorophyll}\;b\;\left(\mathrm{mg}\cdot {\mathrm{mL}}^{-1}\right)=18.61{\mathrm{OD}}_{645}-3.960{\mathrm{OD}}_{662}.$$

(3) $$\mathrm{Total}\;\mathrm{carotenoids}\;\left(\mu g\cdot {\mathrm{mL}}^{-1}\right)=\frac{\left[1000{\mathrm{OD}}_{470}-2.27\mathrm{Chlorophyll}\;a-81.4\;\mathrm{Chlorophyll}\;b\right]}{227}$$

All samples were determined in duplicate, with chlorophyll and carotenoid contents expressed as milligram per gram dried biomass (mg·g^−1^) and microgram per gram dried biomass (µg·g^−1^), respectively.

### 2.6. Phycobiliprotein Determination

Optical densities of the extracted samples from sequential phycobiliprotein extraction were measured at 562, 615 and 652 nm using a UV–Vis spectrophotometer (SP-8001, UV–Vis Spectrophotometer, Metertech, Taipei, Taiwan). Concentrations of C-PC, APC and PE were combined as total phycobiliprotein concentration according to the following equations [60]:

(4) $$C-\mathrm{PC}\;\left(\mathrm{mg}\cdot {\mathrm{mL}}^{-1}\right)=\frac{{\mathrm{OD}}_{615}-0.474{\mathrm{OD}}_{652}}{5.34}.$$

(5) $$\mathrm{APC}\;\left(\mathrm{mg}\cdot {\mathrm{mL}}^{-1}\right)=\frac{{\mathrm{OD}}_{652}-0.208{\mathrm{OD}}_{615}}{5.09}.$$

(6) $$\mathrm{PE}\;\left(\mathrm{mg}\cdot {\mathrm{mL}}^{-1}\right)=\frac{{\mathrm{OD}}_{562}-2.41C-\mathrm{PC}-0.849\mathrm{APC}}{9.62}.$$

where C-PC, APC and PE are C-phycocyanin, allophycocyanin and phycoerythrin concentration. All samples were determined in duplicate, with phycobiliprotein concentration expressed as milligrams per millilitre (mg·mL^−1^) and phycobiliprotein extraction yields expressed as milligrams per gram of dried biomass (mg·g^−1^).

(7) $$C-\mathrm{PC}\;\mathrm{Yield}\;\left(\mathrm{mg}\cdot g^{-1}\right)=\frac{C-\mathrm{PC}*V}{\mathrm{Dried}\;\mathrm{Biomass}}.$$

(8) $$\mathrm{APC}\;\mathrm{Yield}\;\left(\mathrm{mg}\cdot g^{-1}\right)=\frac{\mathrm{APC}*V}{\mathrm{Dried}\;\mathrm{Biomass}}$$

(9) $$\mathrm{PE}\;\mathrm{Yield}\;\left(\mathrm{mg}\cdot g^{-1}\right)=\frac{\mathrm{PE}*V}{\mathrm{Dried}\;\mathrm{Biomass}}.$$

### 2.7. Extract Purity

Extract purity of the phycobiliproteins was determined according to the absorbance at 562, 615, 652 and 280 nm using a UV–Vis spectrophotometry (SP-8001, UV–Vis Spectrophotometer, Metertech, Taipei, Taiwan) according to the following equations [60]:

(10) $$C-\mathrm{PC}\;=\frac{{\mathrm{OD}}_{615}}{{\mathrm{OD}}_{280}}.$$

(11) $$\mathrm{APC}\;=\frac{{\mathrm{OD}}_{652}}{{\mathrm{OD}}_{280}}.$$

(12) $$\mathrm{PE}\;=\frac{{\mathrm{OD}}_{562}}{{\mathrm{OD}}_{280}}.$$

The purities of C-phycocyanin (C-PC), allophycocyanin (APC) and phycoerythrin (PE) fractions were calculated using the ratios of absorbance at 615, 652 and 562 divided by 280 nm, while absorbance at 280 nm revealed total protein concentration in the extracted samples [62].

### 2.8. Total Phenolic Content

Total phenolic content of the extracted samples from sequential phycobiliprotein extraction was determined using the Folin–Ciocâlteu colourimetric method with slight modifications [14]. Briefly, 20 μL of the sample was mixed with 100 µL of 0.2 N Folin–Ciocâlteu solution (SRL, Mumbai, India) and 80 µL of 0.7 M sodium carbonate solution, followed by incubation at room temperature for 8 min. Then, 50 μL of distilled water was added to the mixture, before incubating at 40 °C for 30 min. The absorbance was measured at 750 nm using a microplate reader (M965+, Microplate Reader, Metertech, Taipei, Taiwan). Gallic acid was used as the standard. All samples were determined in duplicate, with results expressed as mg gallic equivalent (mg GA·g^−1^).

### 2.9. ABTS Assay

The ABTS radical-scavenging antioxidant activity of the extracted samples from sequential phycobiliprotein extraction was determined following a previously described method [63] with slight modifications. Briefly, the ABTS radical solution was prepared from the reaction between 505.05 µL of 7 mM ABTS (2,2-azino-bis (3-ethaylbenzothiazoline-6-sulphonic acid) diammonium salt) (SRL, Mumbai, India) and 5.05 µL of 245 mM ammonium persulphate. The mixture was kept in the dark at room temperature for 16 h and then diluted with distilled water to an optical density of 0.7 at 750 nm. Then, 10 μL of sample was mixed with 190 µL of ABTS solution. The mixture was kept in the dark for 5 min. The absorbance was measured at 750 nm using a microplate reader. Ascorbic acid (Sigma-Aldrich, Singapore) was used as the antioxidant standard. All samples were determined in duplicate, with antioxidant capacity expressed as mg ascorbic acid equivalent (mg vitamin C·g^−1^).

### 2.10. FRAP Assay

The ferric ion reducing antioxidant power assay of the extracted samples from sequential phycobiliprotein extraction was determined according to the method of Renugadevi et al. [64] with slight modifications. Briefly, the reagent was prepared from 300 mM sodium acetate (pH 3.6) and 10 mM TPTZ (2,4,6-tris (2-pyridyl)-*s*-triazine) (SRL, India) in 40 mM HCl and 20 mM ferric chloride (Sigma-Aldrich, Singapore) at volumes of 25, 2.5 and 2.5 mL respectively. Then, 10 μL of sample was mixed with 190 µL of FRAP reagent before incubating in the dark for 30 min. The absorbance was measured at 593 nm using a microplate reader. Ascorbic acid (Sigma-Aldrich, Singapore) was used as the standard. All samples were determined in duplicate, with results expressed as mg ascorbic acid equivalent (mg vitamin C·g^−1^).

### 2.11. Statistical Analysis

All parameters from the experiments were statistically analysed by one-way analysis of variance (ANOVA) using SPSS 12.0 (SPPS, Inc., Armonk, NY, USA). Multiple comparisons in all experiments were conducted using Duncan’s multiple range test (DMRT) with a significance level of 0.05.

## 3. Results and Discussion

### 3.1. Supercritical Fluid Extraction (SFE)

**Figure 2:**
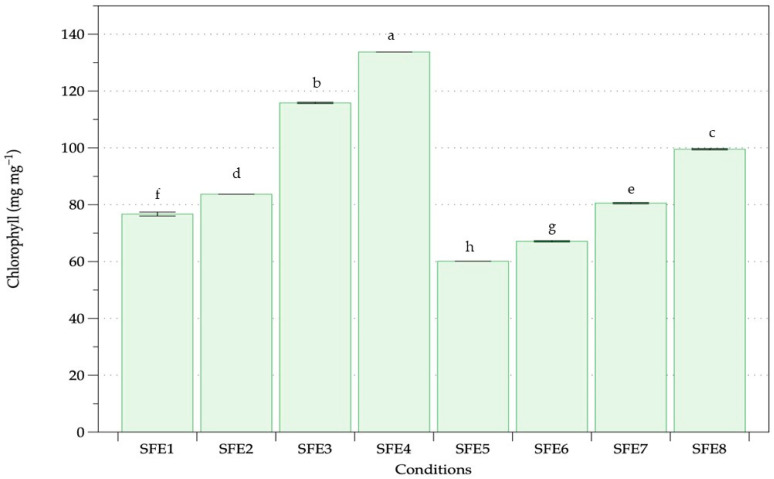
Chlorophyll content of *A. platensis* following SFE under various conditions. Different letters indicate significant differences (*p* < 0.05). Data were calculated from duplicate experimental values ± standard deviation (SD). Abbreviations of conditions of SFE (SFE1–SFE8) are shown in Table 1.

**Figure 3:**
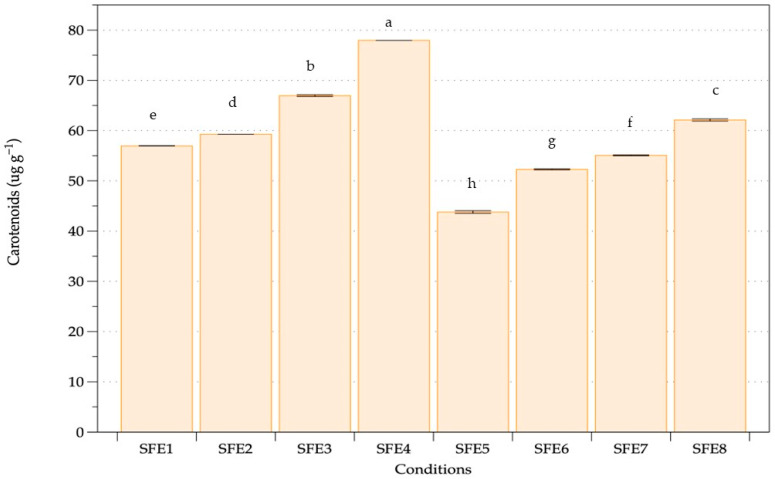
Carotenoid content of *A. platensis* following SFE under various conditions. Different letters indicate significant differences (*p* < 0.05). Data were calculated from duplicate experimental values ± standard deviation (SD). Abbreviations of conditions of SFE (SFE1–SFE8) are shown in Table 1.

Nonpolar pigment extraction including chlorophyll and carotenoids of *A. platensis* oven-dried biomass was performed with various pressure, temperature and cosolvent assistance (Table 1). Two main groups without (SFE1–SFE4) and with (SFE5–SFE8) ethanol as a cosolvent were compared. The chlorophyll content of *A. platensis* using SFE under different conditions is shown in Figure 2. The extraction results revealed a chlorophyll content range of 60.12–133.72 mg·mg^−1^ of dry weight biomass. Higher pressure and temperature achieved higher chlorophyll content both with and without ethanol. The highest amount of chlorophyll extracted was 133.73 mg·mg^−1^ obtained from 350 bar, 50 °C and without ethanol. The carotenoid contents of *A. platensis* using SFE under various conditions are shown in Figure 3, with a range of 43.79–77.95 μg·g^−1^ of dry weight biomass. The highest amount of carotenoid extracted was obtained at the highest pressure and temperature of SC-CO_2_ without ethanol. Higher carotenoid content was observed when increasing the pressure and temperature of extraction both with and without ethanol as a co-solvent. Equal pressure and temperature extraction without ethanol showed higher chlorophyll and carotenoid extraction than with ethanol.

SFE at high pressure and temperature without cosolvent was evaluated for extraction of chlorophylls and carotenoids from *Arthrospira*. Increasing temperature at a constant pressure gave higher chlorophyll and carotenoid extraction, whereas increasing pressure at a constant temperature gave higher chlorophyll and carotenoid. Both pressure and temperature were influencing parameters using SC-CO_2_. The results concurred with previous studies of microalgae and seaweed extraction performed using SC-CO_2_, where higher pressure gave higher extraction and faster kinetic extraction [65]. At higher pressure, the enhancement of carbon dioxide density improved the extraction process with enhanced solubility, while constant temperature and increasing pressure increased yield with faster kinetic extraction due to the relationship between pressure and density [66]. Temperature plays an important role in the SFE process [67]. Pigment extraction is dependent on a delicate equilibrium between the reduction in supercritical carbon dioxide density and the increase in pigment vapour pressure as the temperature rises, essentially representing pigment solubility in the solvent [68]. Higher temperatures assisted higher solute solubility, hence boosting solute mass transfer in the matrix. Our findings were similar to previous results. High pressure and temperature of SFE at 450 bar and 60 °C gave highest carotenoids from *A. platensis* as a suitable green extraction technology [59].

Ethanol at 10% co-solvent did not affect chlorophyll and carotenoid extraction from *A. platensis*. Increasing ethanol percentage increased the extraction and yield of more polar compounds [69]. Nonpolar extracts are generally used to remove or extract nonpolar substances from biomass as unwanted glycosides and lipids [70]. Previous studies revealed that the optimal yield of chlorophylls and carotenoids from *Nannocholopsis gaditana*, *Synechococcus* sp. and *Dunaliella salina* was obtained using SFE with ethanol as a cosolvent compared with the conventional method (methanol extraction) [71]. SFE has various advantages over conventional extraction methods using hexane, petroleum ether, chloroform, ethanol and methanol to recover nonpolar biosubstances from algae [72]. Higher carotenoid and chlorophyll extraction from *Dunaliella salina* was obtained using SFE than by ultrasound-assisted extraction. [73]. Carotenoid extraction from *Chlorella vulgaris* was obtained using SC-CO_2_ fluid extraction under 350 bar and 40 °C and was more difficult than hydrocarbon extraction [74]. The highest yield of pigment depended on the microalgal type, cultivation procedure and other factors.

### 3.2. Sequential Phycobiliprotein Extraction

Previous studies on PBP extraction from *Arthrospira* revealed the optimised conditions to be sonication-assisted extraction with incubation at 25 °C for 24 h [60]. PBPs were extracted under various conditions, with concentration, extraction yield and extract purity shown in Table 2, Table 3 and Table 4. Phosphate buffer resulted in a higher yield of PBPs than water. C-PC, APC, PE and PBP concentration ranges were 0.57–1.17, 0.08–0.31, 0.03–0.09 and 0.67–1.55 mg·mL^−1^, respectively (Table 2). Highest concentration of C-PC was achieved for SFE4 using phosphate buffer but was not significantly different from SFE1–SFE3. Highest concentrations of APC and PE were achieved for SFE1 using phosphate buffer. The highest PBP concentration was achieved for SFE4. In general, greater PBP yields were obtained following SFE without ethanol cosolvent assistance (SFE1-4). The control experiments showed the lowest parameters in all cases. Table 3 shows the yield of PBP extraction under various conditions. The yield ranges of C-PC, APC, PE and PBP were 29.18–56.09, 4.05–14.55, 1.31–4.46 and 34.54–73.40 mg·g^−1^, respectively. The SFE4 condition with phosphate buffer extraction provided the highest C-PC yield, whereas the SFE1 condition with phosphate buffer extraction gave the highest APC and PE yields. The highest PBP yield was achieved for the SFE4 condition with phosphate buffer extraction. The control experiments showed the lowest extract yield. Table 3 shows the extract purity of the phycobiliprotein extraction from *A. platensis* SFE residues. The maximum extract purities of C-PC, APC and PE were 0.61, 0.24 and 0.30, respectively, following phosphate buffer extraction. C-PC extracted under the SFE5 condition with phosphate buffer showed maximum extract purity, which was non-significantly different from SFE1 with phosphate buffer extraction. The control experiments resulted in the lowest extract purity. The sequential extraction of phycobiliproteins from *Arthrospira* cell residues following SFE led to improvement in concentration, extraction yield and extract purity compared to the control.

For *A. platensis* residues prepared from SFE without ethanol as cosolvent, PBP concentration and extraction yield were higher than ethanol-assisted extraction. All experiments achieved higher C-PC, APC, PE and PBP concentration and extraction yield than the control experiment (*A. platensis* without SFE). PBPs are a complex group containing C-PC, APC and PE as the major classes of water-soluble pigments [75]. The SFE process with CO_2_ separated the nonpolar pigment. Hence, *A. platensis* residues from SC-CO_2_ remained mainly as PBP water-soluble pigments with fewer nonpolar contaminants. The variable pressure and temperature conditions of the SFE process of *A. platensis* did not affect PBP extraction from *A. platensis* biomass residues. Our results showed that phycobiliproteins extracted after SFE without ethanol showed a higher yield of phycobiliproteins. Previous results showed the yields of C-PC, APC, PE and PBPs without the SFE process to be 43.57, 2.59, 3.60 and 40.72 mg g^−1^, respectively. Thus, our results showed higher PBPs from *A. platensis* residues in the SFE process. Water can be used as a green extraction solvent [76]. Both chlorophyll and carotenoid extraction of *A. platensis* with SC-CO_2_ and PBP water extraction of *A. platensis* SFE residues were enhanced using green technologies and a sustainable extraction process. Previous studies of water extraction of PBPs from marine *Spirulina maxima* using ultrasonication extraction at 20–100 kHz achieved a high concentration and yield of PBP extraction [77]. High levels of proteins were also found in PBPs from *Arthrospira* [34]. One of the key factors influencing aggregation and dissociation to produce monomers, trimers, hexamers and other oligomers in solution is the pH value. Trimers were produced from C-PC with highest solubility at pH 7.0 [78]. Our results showed that concentration and yield of PBPs under phosphate buffer extraction were higher than using water extraction. PBPs are extracted more effectively due to their enhanced solubility and diffusion rate at pH 7.0 maintained under phosphate buffer. When utilising diluted phosphate buffer for extraction, the osmotic shock may result in cell wall rupture [38]. Therefore, our results confirmed the optimal condition of PBP extraction using cell residues from SFE, as well as the suitability of phosphate buffer for extraction.

**Figure 4:**
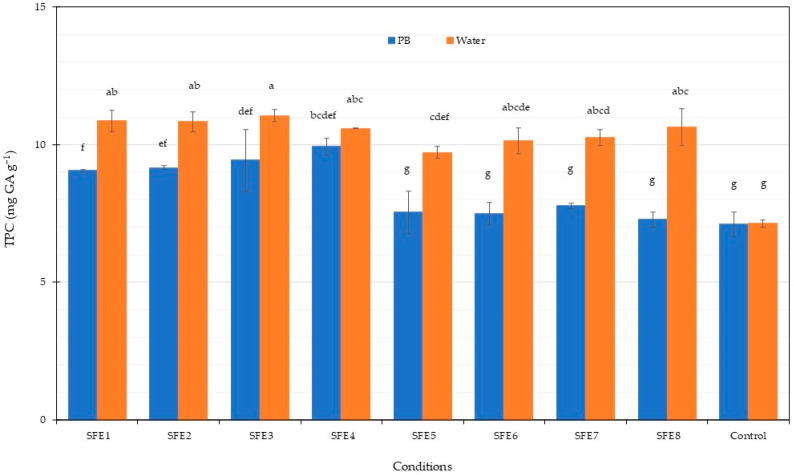
Total phenolic content of *A. platensis* SFE residues under various conditions. Different letters indicate significant differences (*p* < 0.05). Data were calculated from triplicate experimental values ± standard deviation (SD). PB and water are phosphate and water extraction. The abbreviations of conditions of SFE (SFE1-SFE8) are shown in Table 1.

**Figure 5:**
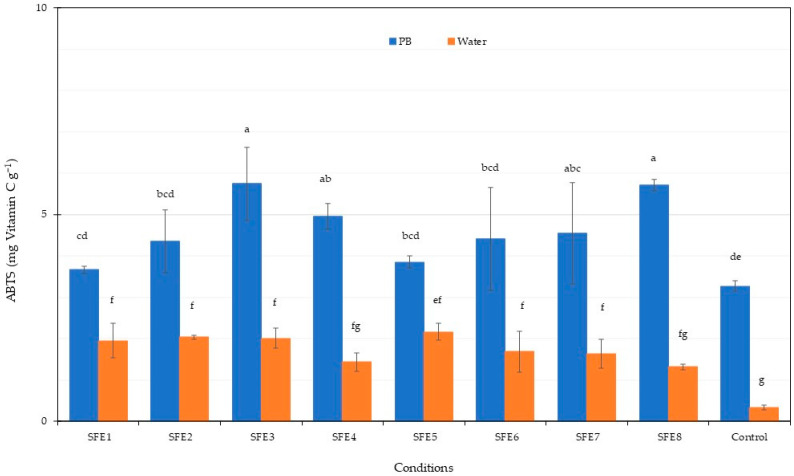
ABTS radical scavenging activity of *A. platensis* SFE residues under various conditions. Different letters indicate significant differences (*p* < 0.05). Data were calculated from triplicate experimental values ± standard deviation (SD). PB and water are phosphate and water extraction. The abbreviations of conditions of SFE (SFE1–SFE8) are shown in Table 1.

**Figure 6:**
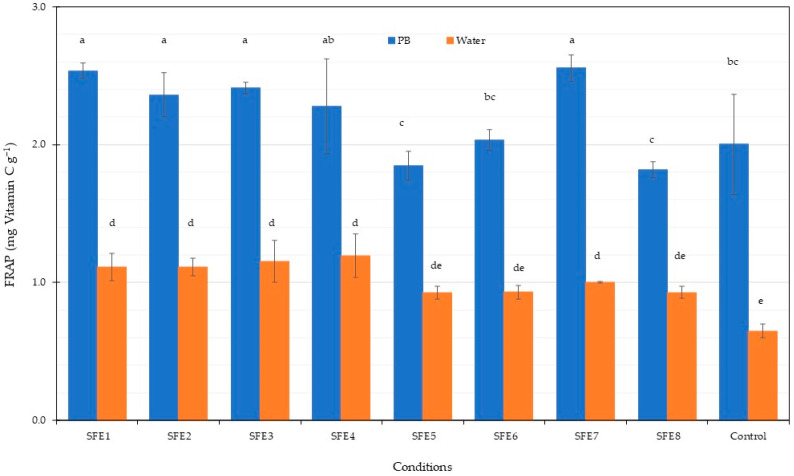
Ferric reducing antioxidant power assay of *A. platensis* SFE residues under various conditions. Different letters indicate significant differences (*p* < 0.05). Data were calculated from triplicate experimental values ± standard deviation (SD). PB and water are phosphate and water extraction. The abbreviations of conditions of SFE (SFE1–SFE8) are shown in Table 1.

The total phenolic content in the PBP extract from *Arthrospira* after SFE is shown in Figure 4. All experiments with water gave higher TPC compared to the phosphate buffer (PB). A TPC of approximately 10 mg·g^−1^ was obtained from water extraction. The control experiments gave the lowest TPC in all cases. Previous results found that water extraction from *Stypocaulon scoparium* algae gave the highest TPC among several solvents including water-methanol, methanol and ethanol [79]. Therefore, water was a suitable solvent for total phenolic content extraction. The antioxidant potential of the PBP extract from *Arthrospira* residues after SFE was assessed using ABTS and FRAP methodologies, with results shown in Figure 5 and Figure 6. ABTS and FRAP antioxidant assay involves a single electron transfer process; however, the ABTS is distinguished by antioxidant reducing power, which is measured by the ability to reduce a coloured stable free radical (ABTS•+), while FRAP is distinguished by the antioxidant chemical ability to reduce Fe^3+^ ions to blue Fe^2+^ ions [80]. The ABTS radical-scavenging activity of the PBP extract was higher following phosphate buffer extraction compared to water extraction. Maximum ABTS at 5.7 mg·g^−1^ was achieved for the SFE3 and SFE8 conditions. The FRAP antioxidant activities of the PBP extract from *Arthrospira* residues after SFE were higher following phosphate buffer extraction compared to water extraction, ranging from 1.82 to 2.56 mg·g^−1^. Our results indicated that PBP extracts contained phenolic compounds and exhibited antioxidant capacity. Antioxidant activity is not only caused by phenolic substances [79]. C-PC blue colourant was observed from *A. platensis* SFE residues as the main pigment. Therefore, antioxidant properties in our results were caused by PBP extraction from *A. platensis.* Higher PBP extraction was exhibited from phosphate buffer extraction and the assay also gave higher antioxidant activity. Previous results showed the antioxidant activities of various C-PC concentrations from *A. platensis* using the ABTS assay [81]. The scavenging capacity of ABTS increased concentration dependently [82]. The antioxidant properties of C-PC can be used as a food supplement. The previous study of C-PC purified by ultrafiltration presented the antioxidant activity against the ABTS 206.36 µmol Trolox g^−1^ of ice cream with C-PC incorporated increasing the antioxidant activity after digestion [63]. Data from both ABTS and FRAP assays showed that PBPs had significant antioxidant properties and could be considered as food for human health improvement. C-PC showed high antioxidant activity which could be applied in several sectors. Reactive oxygen species (ROS) and oxidative processes are recognised as playing an ameliorating role in a number of illnesses including atherosclerosis, diabetes and Alzheimer’s disease [82].

### 3.3. C-Phycocyanin Isolation

**Figure 7:**
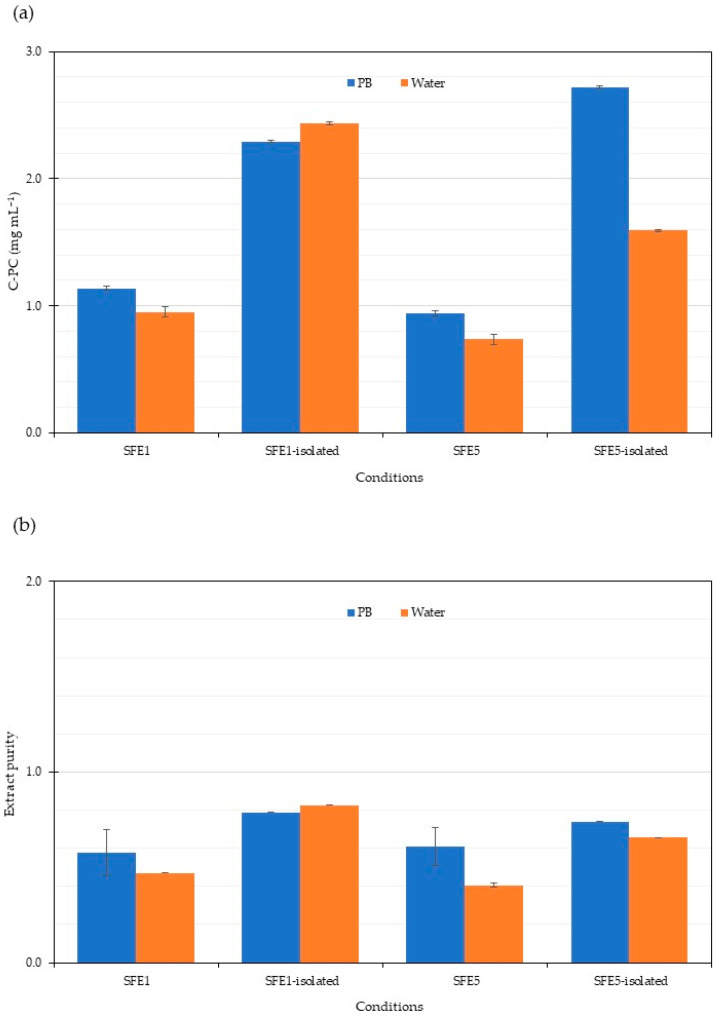
Purity of C-PC isolated from *A. platensis* SFE residues. Data were calculated from triplicate experimental values ± standard deviation (SD). PB and water are phosphate and water extraction. (**a**) C-PC concentration and (**b**) Extract purity of C-PC isolated from *A. platensis* SFE residues

C-PC is the main PBP extracted from *Arthrospira*. Price and C-PC quality are directly correlated, with higher cost corresponding to a purer product [83]. Crude C-phycocyanin supernatants from SFE1 and SFE5 cell residues following phosphate and water extraction were selected to improve C-PC purity by ultrafiltration. C-PC concentration and purity (Figure 7) revealed an increase to approximately 0.7, similar to other purification methods. Previous results from several steps of purification of C-PC were studied. The purification of C-PC using activated charcoal for 24 h gave the highest purity of 1.2 [60]. Our results in this study used a short time for purification of 15 min. Crude C-PC isolated using ammonium sulphate precipitation followed by ion exchange chromatography gave higher purity than our results [84]. Crude C-PC showed improved purity for ammonium sulphate precipitation, ultrafiltration, gel filtration, and ion exchange chromatography [85]. The purification procedures follow several steps to attain high purity of C-PC as a valuable high cost bioproduct.

## 4. Conclusions

*A. platensis* is a commercially available blue-green microalga that is used as a food source for human health. Supercritical fluid extraction (SFE) was successfully applied to obtain the chlorophylls, carotenoids and phycobiliproteins remaining in cell residues. The optimal yield of nonpolar biocompounds (chlorophylls and carotenoids) was achieved using SFE at high pressure and temperature. Ethanol as a cosolvent did not improve extraction during the SC-CO_2_ process. The optimal yield of phycobiliproteins from SFE residues was achieved using a phosphate buffer extraction without cosolvent, while the purity of C-phycocyanin (C-PC) was improved.

## Figures and Tables

**Table 1:** Experimental conditions for supercritical fluid extraction (SFE) of *A. platensis*.

Experiment	Pressure	Temperature	Cosolvent
	(bar)	(°C)	(% *w*/*w*)
SFE1	250	40	None
SFE2	250	50	None
SFE3	350	40	None
SFE4	350	50	None
SFE5	250	40	10% ethanol
SFE6	250	50	10% ethanol
SFE7	350	40	10% ethanol
SFE8	350	50	10% ethanol

**Table 2:** Phycobiliprotein concentration of *A. platensis* SFE residues under various conditions. Data in the same column with different superscripts are significantly different (*p* < 0.05). Data were calculated from triplicate experimental values ± standard deviation (SD). C-PC, APC, PE and PBP are C-phycocyanin, allophycocyanin, phycoerythrin and total phycobiliprotein concentration. The abbreviations of conditions of SFE (SFE1–SFE8) are shown in Table 1.

Experiment	Phycobiliprotein Concentration (mg·mL^−1^)			
	C-PC	APC	PE	PBP
Phosphate buffer extraction				
SFE1	1.139 ^a^ ± 0.02	0.307 ^a^ ± 0.01	0.094 ^a^ ± 0.00	1.540 ^a^ ± 0.03
SFE2	1.140 ^a^ ± 0.03	0.298 ^ab^ ± 0.01	0.093 ^ab^ ± 0.01	1.530 ^a^ ± 0.01
SFE3	1.156 ^a^ ± 0.02	0.275 ^b^ ± 0.01	0.082 ^c^ ± 0.00	1.513 ^a^ ± 0.02
SFE4	1.174 ^a^ ± 0.03	0.291 ^ab^ ± 0.01	0.085 ^bc^ ± 0.01	1.550 ^a^ ± 0.04
SFE5	0.945 ^b^ ± 0.02	0.200 ^c^ ± 0.01	0.062 ^d^ ± 0.00	1.207 ^b^ ± 0.03
SFE6	0.886 ^bcd^ ± 0.01	0.184 ^cd^ ± 0.01	0.059 ^de^ ± 0.00	1.129 ^bcd^ ± 0.03
SFE7	0.959 ^b^ ± 0.07	0.207 ^c^ ± 0.02	0.065 ^d^ ± 0.01	1.231 ^b^ ± 0.10
SFE8	0.860 ^bcde^ ± 0.09	0.189 ^cd^ ± 0.02	0.060 ^d^ ± 0.01	1.109 ^bcd^ ± 0.12
Control	0.766 ^ef^ ± 0.09	0.142 ^f^ ± 0.01	0.045 ^f^ ± 0.00	0.953 ^ef^ ± 0.11
Water extraction				
SFE1	0.953 ^b^ ± 0.04	0.193 ^c^ ± 0.01	0.059 ^de^ ± 0.00	1.205 ^b^ ± 0.06
SFE2	0.960 ^b^ ± 0.02	0.200 ^c^ ± 0.01	0.063 ^d^ ± 0.00	1.223 ^b^ ± 0.02
SFE3	0.911 ^bc^ ± 0.07	0.196 ^c^ ± 0.01	0.064 ^d^ ± 0.00	1.171 ^bc^ ± 0.07
SFE4	0.957 ^b^ ± 0.03	0.196 ^c^ ± 0.01	0.060 ^d^ ± 0.00	1.214 ^b^ ± 0.03
SFE5	0.737 ^f^ ± 0.04	0.135 ^f^ ± 0.01	0.043 ^f^ ± 0.00	0.915 ^f^ ± 0.05
SFE6	0.788 ^def^ ± 0.00	0.148 ^ef^ ± 0.00	0.046 ^f^ ± 0.00	0.982 ^ef^ ± 0.01
SFE7	0.839 ^cde^ ± 0.00	0.167 ^de^ ± 0.00	0.051 ^ef^ ± 0.00	1.056 ^cde^ ± 0.01
SFE8	0.806 ^def^ ± 0.01	0.155 ^ef^ ± 0.00	0.049 ^f^ ± 0.00	1.011 ^def^ ± 0.01
Control	0.568 ^g^ ± 0.02	0.079 ^g^ ± 0.01	0.025 ^g^ ± 0.00	0.672 ^g^ ± 0.03

**Table 3:** Phycobiliprotein extraction yield from *A. platensis* SFE residues under various conditions. Data in the same column with different superscripts are significantly different (*p* < 0.05). Data were calculated from triplicate experimental values ± standard deviation (SD). C-PC, APC, PE and PBP are C-phycocyanin, allophycocyanin, phycoerythrin and total phycobiliprotein. The abbreviations of conditions of SFE (SFE1–SFE8) are shown in Table 1.

Experiments	Phycobiliprotein Extraction Yield (mg·g^−1^)			
	C-PC	APC	PE	PBP
Phosphate buffer extraction				
SFE1	54.072 ^a^ ± 0.93	14.550 ^a^ ± 0.01	4.460 ^a^ ± 0.05	73.082 ^a^ ± 0.98
SFE2	54.486 ^a^ ± 2.27	14.216 ^ab^ ± 0.15	4.417 ^a^ ± 0.40	73.119 ^a^ ± 1.72
SFE3	56.091 ^a^ ± 0.73	13.325 ^b^ ± 0.66	3.988 ^b^ ± 0.27	73.404 ^a^ ± 1.66
SFE4	55.021 ^a^ ± 0.27	13.636 ^ab^ ± 0.05	3.985 ^b^ ± 0.04	72.643 ^a^ ± 0.17
SFE5	46.864 ^bc^ ± 1.20	9.926 ^c^ ± 0.45	3.053 ^c^ ± 0.15	59.843 ^bc^ ± 1.80
SFE6	43.038 ^cde^ ± 0.84	8.930 ^cd^ ± 0.13	2.873 ^cd^ ± 0.04	54.841 ^cde^ ± 0.76
SFE7	46.428 ^bc^ ± 2.84	10.015 ^c^ ± 1.02	3.160 ^c^ ± 0.45	59.603 ^bc^ ± 4.31
SFE8	41.206 ^de^ ± 2.90	9.034 ^cd^ ± 0.92	2.874 ^cd^ ± 0.18	53.114 ^def^ ± 3.99
Control	38.965 ^ef^ ± 4.65	7.227 ^f^ ± 0.67	2.300 ^e^ ± 0.02	48.492 ^fg^ ± 5.35
Water extraction				
SFE1	46.945 ^bc^ ± 2.25	9.525 ^c^ ± 0.57	2.897 ^cd^ ± 0.09	59.367 ^bc^ ± 2.91
SFE2	47.703 ^b^ ± 0.94	9.924 ^c^ ± 0.35	3.139 ^c^ ± 0.07	60.765 ^b^ ± 1.36
SFE3	45.344 ^bcd^ ± 3.01	9.770 ^c^ ± 0.36	3.203 ^c^ ± 0.01	58.316 ^bcd^ ± 3.37
SFE4	47.462 ^bc^ ± 1.46	9.706 ^c^ ± 0.28	2.998 ^c^ ± 0.03	60.167 ^bc^ ± 1.71
SFE5	36.610 ^f^ ± 2.00	6.710 ^f^ ± 0.44	2.145 ^e^ ± 0.08	45.465 ^g^ ± 2.52
SFE6	39.238 ^ef^ ± 0.19	7.349 ^ef^ ± 0.03	2.302 ^e^ ± 0.10	48.889 ^fg^ ± 0.33
SFE7	41.770 ^de^ ± 0.12	8.303 ^de^ ± 0.11	2.521 ^de^ ± 0.07	52.594 ^ef^ ± 0.30
SFE8	40.135 ^ef^ ± 0.16	7.734 ^ef^ ± 0.02	2.463 ^e^ ± 0.12	50.333 ^efg^ ± 0.30
Control	29.180 ^g^ ± 1.14	4.052 ^g^ ± 0.33	1.306 ^f^ ± 0.14	34.539 ^h^ ± 1.61

**Table 4:** Extract purity of *A. platensis* SFE residues under various conditions. Data in the same column with different superscripts are significantly different (*p* < 0.05). Data were calculated from triplicate experimental values ± standard deviation (SD). C-PC, APC and PE are C-phycocyanin allophycocyanin and phycoerythrin. The abbreviations of conditions of SFE (SFE1-SFE8) are shown in Table 1.

Experiments	Extract Purity		
	C-PC	APC	PE
Phosphate buffer extraction			
SFE1	0.578 ^ab^ ± 0.12	0.240 ^a^ ± 0.05	0.299 ^ab^ ± 0.06
SFE2	0.472 ^cd^ ± 0.02	0.193 ^bcde^ ± 0.01	0.243 ^cde^ ± 0.02
SFE3	0.498 ^bcd^ ± 0.01	0.195 ^bcd^ ± 0.01	0.250 ^bcd^ ± 0.01
SFE4	0.536 ^abc^ ± 0.06	0.214 ^abc^ ± 0.03	0.270 ^abc^ ± 0.03
SFE5	0.611 ^a^ ± 0.10	0.228 ^ab^ ± 0.04	0.303 ^a^ ± 0.05
SFE6	0.412 ^d^ ± 0.00	0.153 ^defg^ ± 0.00	0.205 ^de^ ± 0.00
SFE7	0.485 ^bcd^ ± 0.01	0.183 ^cdef^ ± 0.00	0.242 ^cde^ ± 0.00
SFE8	0.398 ^d^ ± 0.06	0.151 ^efg^ ± 0.03	0.200 ^de^ ± 0.03
Control	0.418 ^d^ ± 0.00	0.148 ^fg^ ± 0.00	0.204 ^de^ ± 0.01
Water extraction			
SFE1	0.470 ^cd^ ± 0.00	0.173 ^cdef^ ± 0.00	0.231 ^cde^ ± 0.00
SFE2	0.473 ^cd^ ± 0.01	0.176 ^cdef^ ± 0.01	0.235 ^cde^ ± 0.01
SFE3	0.448 ^cd^ ± 0.03	0.169 ^defg^ ± 0.01	0.226 ^cde^ ± 0.01
SFE4	0.497 ^bcd^ ± 0.01	0.183 ^cdef^ ± 0.00	0.245 ^cd^ ± 0.01
SFE5	0.407 ^d^ ± 0.01	0.144 ^fg^ ± 0.01	0.199 ^de^ ± 0.01
SFE6	0.414 ^d^ ± 0.01	0.148 ^fg^ ± 0.00	0.202 ^de^ ± 0.00
SFE7	0.419 ^d^ ± 0.02	0.153 ^defg^ ± 0.01	0.205 ^de^ ± 0.01
SFE8	0.434 ^cd^ ± 0.01	0.156 ^defg^ ± 0.00	0.213 ^de^ ± 0.00
Control	0.405 ^d^ ± 0.01	0.130 ^g^ ± 0.00	0.190 ^e^ ± 0.00

## Data Availability

Not applicate.
